# The American Illustrated Medical Dictionary

**Published:** 1902-12

**Authors:** 


### Reviews of Books.

---

**The American Illustrated Medical Dictionary.** By W. A. NEWMAN DORLAND, M.D. Pp. 770. London and Philadelphia: W. B. Saunders & Company. 1900.

The English-speaking practitioner has no ground for complaining that his philological needs are neglected. On both sides of the Atlantic medical dictionaries are issued with almost perplexing frequency, varying from the traditional "vest-pocket" booklet to the portly volume for which it is difficult to find accommodation on an ordinary shelf. In size Dr. Dorland's compilation occupies an intermediate position, and ought to be extremely popular, not only for that reason, but also for its general trustworthiness. It is more than a mere word-book, often giving an account in detail of the symptoms or signs of a disease. The "anatomic" tables, as Dr. Dorland calls them, are specially useful, and are embellished by some very helpful coloured plates. Many of the other subjects are elucidated by appropriate and sufficient illustrations, coloured and otherwise. Bacteriology in its various aspects is very fully considered. The work, presented in a very attractive dress, reflecting great credit upon printer and publisher, is one the purchase of which no doctor will ever regret.

